# Understanding the interplay between cognitive changes at menopause, the workplace, and mental health: qualitative findings from the MARIE-UK (WP2a) project

**DOI:** 10.3389/fgwh.2026.1878423

**Published:** 2026-10-09

**Authors:** Sharron Hinchliff, Kristina Potočnik, Gayathri Delanerolle, Julie Taylor, Kathryn Elliot, Donatella Fontana, Lucky Saraswat, Nick Panay, Vikram Talaulikar, Carol Atkinson, Helen Felicity Kemp, Om Kurmi, Nirmala Rathnayake, Lamya Al-Kharusi, Vindya Pathiraja, Nihal Al-Riyami, Ramya Palanisamy, George Uchenna Eleje, Peter Phiri

**Affiliations:** 1 University of Sheffield, Sheffield, United Kingdom; 2 University of Edinburgh, Edinburgh, United Kingdom; 3 Hampshire and Isle of Wight Healthcare NHS Foundation Trust, Southampton, United Kingdom; 4 University of Birmingham, Birmingham, United Kingdom; 5 University of Aberdeen, Aberdeen, United Kingdom; 6 Imperial College Healthcare NHS Trust, London, United Kingdom; 7 University College London Hospitals NHS Foundation Trust, London, United Kingdom; 8 Manchester Metropolitan University, Manchester, United Kingdom; 9 Trauma Healing Together, Perth, United Kingdom; 10 Centre for Healthcare and Communities, Coventry University, Coventry, United Kingdom; 11 University of Ruhuna, Matara, Sri Lanka; 12 Sultan Qaboos University, Muscat, Oman; 13 The Misbourne Surgery, Chalfont St Peter, United Kingdom; 14 Nnamdi Azikiwe University Teaching Hospital, Nnewi, Nigeria

**Keywords:** cognitive performance, menopause, mental health, qualitative, workplace, UK, MARIE

## Abstract

**Background:**

Cognitive changes are widely experienced during the menopause transition and, when severe, can disrupt women's working lives and negatively impact their mental health. While prior research has documented the detrimental effect of menopausal symptoms on women's quality of life, there remains a paucity of empirical evidence specifically exploring the intersection between cognitive changes and identity reconstruction within occupational settings. The objective of this study was to understand women's experiences of cognitive changes at work during menopause, including how they navigate these difficulties.

**Methods:**

This qualitative, exploratory study, involved in-depth interviews with 50 women aged 43–67 years (mean 52.3) recruited for the MARIE-UK (WP2a) project. Data were analysed using collaborative reflexive thematic analysis, through the lens of Conservation of Resources (COR) theory. Data trustworthiness was ensured through investigator consensus.

**Results:**

The majority of participants experienced cognitive changes at menopause, such as brain fog and memory problems, which negatively impacted their mental health at work. Participants experienced loss of confidence in their abilities, an increase in anxiety, and a change in their sense of self. Due to the depletion of valuable resources (resources are anything an individual values), they struggled to regain a semblance of normality. Those who received workplace support managed to break the spiral of resource loss and better cope with the detrimental effects of the symptoms.

**Conclusion:**

Understanding women's experiences of cognitive changes and mental health in the workplace through the COR theory has shown that menopause is a demand that can significantly deplete women's valuable resources to perform their work duties. Menopausal cognitive changes profoundly disrupted their sense of self and perceived ability to work effectively. The intersection of cognitive and psychological symptoms with workplace stressors exacerbated mental health burdens and perpetuated gender inequalities. Interventions to support workplace wellbeing are required, particularly as the proportion of menopausal women in paid employment increases.

## Background

Menopause marks a biopsychosocial transition in a woman's life, often accompanied by physical, emotional, and cognitive changes (1). While the impact of menopausal symptoms on women's lives has been widely studied, attention is now being paid to the mental health challenges they experience during this period, personally and professionally. The experience of menopause is highly individualised, with some reporting mild to moderate symptomatology, while others suffer severe symptoms which have a detrimental effect on their working lives (2). Past research has found that the severity of menopause symptoms is positively associated with burnout (3, 4), and negatively associated with perceived job performance and retention (4–6). As the UK has been facing a significant skills shortage for a number of years, the working population has been encouraged and incentivized to continue working for longer (4, 7). Women's employment rates have steadily improved with evidence showing that 68.6% percent of women between 50 and 64 years of age were employed, an increase of more than 20% since 1992 (8). This is a positive trend, although research has indicated that menopause symptoms typically occur in our 40s (4).

Cognitive experiences during the menopause transition are heterogeneous and occur within a multifactorial biopsychosocial context. Difficulties involving memory, concentration, word retrieval and information processing may coexist with sleep disruption, vasomotor symptoms, pain, fatigue, psychological symptoms, comorbidity, medication use and occupational stress. Although endocrine changes may contribute to some cognitive experiences, this qualitative study did not measure biological mechanisms and does not attribute participants' accounts to a single hormonal or neurological pathway (see Figure 1). Symptoms such as forgetfulness and difficulty concentrating can severely undermine self-confidence, performance, and impact the overall wellbeing of an employee (5). Perimenopausal, menopausal, and post-menopausal experiences comprise physical, physiological, and psychological symptoms that require holistic management (9).

**Figure 1:**
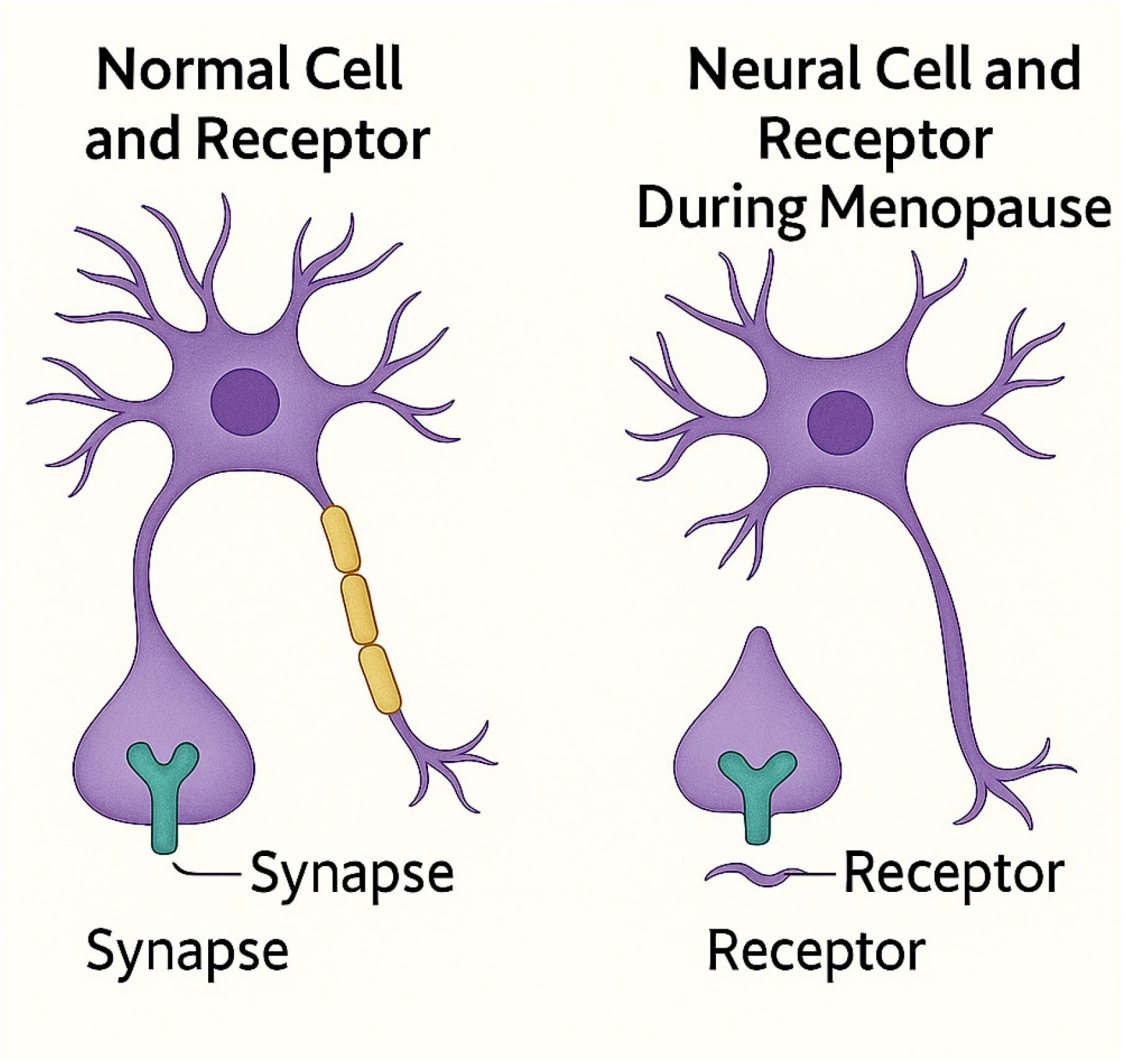
Comparison of a normal neural cell and receptor with a neural cell and receptor during menopause, illustrating changes associated with cognitive symptoms.

The need to provide better support for women during the menopausal transition has been recognised by employers across public, private, and voluntary sectors. Legislations are set to expand in the United Kingdom (UK) with changes to the Employment Rights Bill, where the initial report (July 2025) outlined that menopause action plans will take effect for all employers from 2027. The UK government has outlined a roadmap for delivering these changes as part of the “Implementing the Employment Rights Bill”, emphasising the need for workplace policies and pathways that support employees. Similar actions are being taken elsewhere: for example, the need for specific workplace menopause policies has been recognised in the US, Australia, and Canada.

### Theory

The Conservation of Resources (COR) theory (10) is a useful theory to apply to this topic, as it helps us to understand how menopause symptoms, workplace demands, and mental health can influence each other through the gain and loss of resources. COR enables us to see beyond the biological effects of menopause, to view it in social and interpersonal context, as an experience that can affect an individual's ability to obtain, maintain, and protect the resources they need to function and thrive at work. COR posits that individuals rely on valued resources, such as time, energy, and money, when responding to various demands (resources are anything the individual perceives to be valuable) (11). The use of these resources can lead to their depletion, and such losses are often linked to adverse outcomes, including increased strain and diminished well-being. Individuals should therefore strive to replenish and accumulate their valuable resources so as to experience positive outcomes. Resources are differentiated between personal (e.g., time and health) and contextual (e.g., social support) (12) both of which are conceptualized as “key” resources - i.e., those that help individuals manage their other resources (10). Flexible work arrangements could be considered as a “key” resource because they can help individuals manage their time and energy more efficiently.

COR theory has been used to explore the effects of menopausal physical and psychological symptoms as a type of demand on diverse workplace outcomes, and the role of workplace social support in determining these effects (2, 5). Steffan and Potočnik (2023) observed a negative association of the severity of psychological symptoms with retention, and a negative association of the severity of physical symptoms with perceived performance. They also found that the use of selection, optimization and compensation (SOC) strategies as a type of personal resource, in conjunction with supervisory support as a type of contextual resource, buffered the negative effects of physical symptoms on performance. Drawing from a longitudinal sample, Potočnik et al. (2025) showed how changes in the severity of physical and psychological menopause symptoms over time in the context of work can be associated with either resource depletion (i.e., when symptoms worsen) or accumulation (i.e., when symptoms become less severe), which explained changes in emotional exhaustion and depersonalization – i.e., two dimensions of burnout (2). This study also showed that the usefulness of flexible work – another valuable “key” resource – protected women who experienced more severe physical symptoms over time.

Despite increasing evidence on menopause symptom burden and employment outcomes, less is known about the processes through which cognitive experiences interact with occupational demands, mental health, professional identity, disclosure and workplace support. This paper addresses that experiential and contextual gap within the prospectively designed MARIE-UK WP2a qualitative component.

The aim of this paper is to understand women's experiences of cognitive changes during perimenopause, menopause, and post-menopause, their impact on work performance, and consequences for mental health. The findings are discussed in relation to COR theory to reveal data-driven implications for health and wellbeing as well as workplace support. Understanding women's lived experiences of cognitive symptoms across the menopausal transition is essential to inform targeted interventions, workplace accommodations, and mental health support strategies. This is particularly important given the growing proportion of midlife women in the workforce globally and the economic and societal implications of unaddressed menopausal challenges.

## Methods

This paper reports an exploratory qualitative study embedded within the explanatory sequential mixed-methods design of MARIE-UK WP2a. The wider WP2a project comprised a prospective quantitative cohort followed by qualitative interviews. The present paper concerns the qualitative component, which used in-depth, semi-structured interviews and collaborative reflexive thematic analysis.

The research question was exploratory and was not formulated as a test of COR theory. Themes and subthemes were developed through collaborative reflexive thematic analysis. COR was subsequently applied as an interpretive framework to examine how workplace demands and access to personal, interpersonal and organisational resources shaped participants' experiences. Data were not assigned to predetermined COR categories.

### Project context

The data were collected as part of the MARIE-UK project's work-package 2a (WP2a). The MARIE project is a large-scale global health project addressing unmet needs across perimenopause, menopause, and post-menopause. It includes a diverse sample of participants, including age groups, ethnicities, races, cisgender, LGBTQ+, and natural, medical, and surgical menopause. The MARIE project utilises a mixed-methods, explanatory sequential design to gather quantitative and qualitative data (13). The current paper is concerned with the qualitative data collected from a sub-sample (*n* = 50) of the larger sample of UK women (*n* = 1043). COREQ guidelines were consulted when preparing the manuscript (see Supplementary Appendix 1).

### Recruitment and ethics

Participants were recruited from the 1,043 individuals who completed the MARIE-UK WP2a quantitative component. Approximately 95% consented to potential recontact. A purposive maximum-variation approach was used to select 50 participants, seeking variation in age, menopausal stage, menopause pathway, ethnicity, employment circumstances and reported experiences. All 50 selected participants provided written informed consent and completed an interview; there were no subsequent refusals or withdrawals.

Participants were recruited using digital approaches on Facebook, X, LinkedIn, and NHS Trust websites. In addition, NHS app push notifications, clinics at hospitals and primary care, as well as places of worship such as Hindu temples and Buddhist stupas and Muslim mosques were used. Inclusion criteria were: The wider MARIE WP2a protocol permitted adults aged 18 years or older who met the approved menopause-related eligibility criteria, including individuals experiencing premature, surgical or medical menopause. The minimum age criterion was unrelated to sexual orientation or gender identity. All participants included in the present qualitative analysis self-identified as women and were aged 43–67 years, able to give informed consent, and have access to a smartphone, tablet or computer to be able to complete the survey and qualitative interview online.

The participant information sheet and consent form were provided using the XM Qualtrics platform via a QR code. The study, including the recruitment, consent and interview procedures, received a favourable opinion from the East of England–Cambridge South Research Ethics Committee on 31 August 2023 (REC reference 23/EE/0159). The study was sponsored by Hampshire and Isle of Wight Healthcare NHS Foundation Trust. A risk assessment was completed as part of the standard NHS Sponsorship and HRA approval process, where an escalation pathway was put in place if any of the participants required this. The participant information sheet also had information that showed how to obtain support from the NHS if they required this after their interview.

### Data collection and analysis

Data were collected by in-depth interviews conducted by three women interviewers (GD, SH, JT). The interviews were conducted online through a secure Microsoft Teams platform and lasted between 25 and 90 min. At the start of the interview, participants were debriefed about the aim of project, given the chance to ask questions, and then gave their consent to take part. The interview guide was developed specifically for this study and was based on the existing evidence base and insights from the Women's Health Strategy public consultation (14). The interviews explored menopause symptoms and their management, help-seeking and health care, and experiences of menopause symptoms at work and home. Interviews were transcribed verbatim, and all identifying information was removed.

We conducted a collaborative thematic analysis of all 50 interviews following the procedures outlined by Crick et al. (2025), Delve and Limpaecher (2023), and Braun and Clarke (2006) (15–17). Interviews were transcribed verbatim, direct and indirect identifiers were removed, and pseudonyms were assigned before analysis and publication. Names accompanying quotations are pseudonyms and do not identify participants. Each transcript was analysed in full by a core team (SH, KP, KE, DF) using word-processing programmes. First, the team read through the same transcript, identified their own codes, which we then compared to explore shared understanding. We followed the same procedure for an additional two transcripts each and discussed the codes within the team. Agreement on coding decisions was reached, and the remaining transcripts shared so that each team member analysed their own set of 10–11 transcripts. The lead author (SH) created a coding framework based on the first nine coded transcripts, which we then applied to our further transcripts. We continued to meet to discuss the codes and developing themes, and the coding framework was updated accordingly. Themes were identified based on patterns in the data where differences were also acknowledged. This inductive and systematic approach was rigorous, and the collaborative method enriched the analysis by incorporating the diverse expertise of the researchers involved. Analysis of all 50 interviews resulted in 13 themes with a total of 46 subthemes (Supplementary Appendix 2). This paper is concerned with the themes and subthemes around cognitive changes in the workplace.

### Sample characteristics

Participants were aged between 43 and 67 years with an average of 52.3 years. They self-reported their menopausal status as menopause (*n* = 24), post-menopause (*n* = 14), or perimenopause (*n* = 12). Natural menopause (*n* = 43) was predominantly reported with the remaining experiencing surgical (*n* = 5) or medical (*n* = 2) (see Table 1).

**Table 1:** Sample characteristics.

Characteristic	Number of participants (*n*)
Age	
40–49	16
50–59	30
60–69	4
Menopause status	
Peri-menopause	12
Menopause	24
Post-menopause	14
Relationship status	
Married	31
Divorced	6
Single	6
Cohabiting	5
Civil partnership	1
Other	1
Employment status	
Working full-time	32
Working part-time	7
Retired	6
Working flexible hours	2
Home-maker	2
Unemployed	1
Educational level	
Postgraduate degree	15
Undergraduate degree	17
A levels	11
GCSE	5
No formal education	1
No answer	1
Ethnicity	
White	38
South Asian	5
White other	4
Caribbean	1
Black American	1
Peruvian	1

At the time of data collection, 41 participants were in paid employment: 32 worked full-time, seven part-time and two reported flexible working arrangements. Interviews were arranged at mutually convenient times and conducted from a private location chosen by each participant. Employment status does not indicate that participants were interviewed while undertaking work duties. The main fields they worked in were health care, finance, research, and education. Two participants described their occupation as homemaker, six were retired, and one was unemployed (the seven non-working participants reflected on their working lives during the interview).

Participants described their relationship status as: married (*n* = 31); divorced (*n* = 6); single (*n* = 6); co-habiting (*n* = 5); civil partnership (*n* = 1); and other (*n* = 1). The majority had received University level education (postgraduate *n* = 15, undergraduate *n* = 17), with the remaining receiving A' levels (*n* = 11) or GCSE (*n* = 5). One participant reported no formal education, and another did not answer this question. Most described their ethnicity as: white (*n* = 38), white other (*n* = 4), South Asian (*n* = 5), Caribbean (*n* = 1), Black American (*n* = 1), and Peruvian (*n* = 1).

The sample incorporated variation across menopausal stage, menopause pathway and occupational circumstances but was not intended to be statistically representative of the UK population.

## Results

Cognitive changes that affected executive function had a substantial impact on the women's perceptions of their own work performance, which in turn impacted their mental well-being. These experiences are examined through four key themes: Overall impact, perceived inability to work efficiently; Loss of control and sense of self; Anxiety, struggle, and adaptation; and Masking and workplace support.

### Overall impact: perceived inability to work efficiently

The women described cognitive changes, particularly around memory (working, short term, and episodic) and “brain fog” as creating pronounced challenges at work. The combination of brain fog, characterized by difficulty focusing on tasks or not being able to follow instructions, and problems with memory had a serious impact on the women's perceptions of their own ability to work efficiently.

Mind fog, brain fog, whatever you want to call it, forgetting stuff with people saying, “Oh, can you remember we had that conversation?” No, you know, hearing stuff and not taking it in, all of that is far worse than I ever, ever expected it to be. I know, brain fog is a very common thing talked about with menopause, but I didn't expect it to be this real. (Grace)

Grace was surprised at the intensity of the cognitive changes she experienced, even though she was aware that brain fog was a key symptom of menopause. Similarly, other women reported that their cognitive difficulties could appear very quickly, as if out of nowhere.

It just seemed very rapidly that I didn't have the cognitive skills that I did have. I mean, for a number of years in the job that I had, I worked in it for 20 + years. I would be “Oh, if you want to know policy or you know some information go to Bree because she'll be able to explain it better and she knows all about it”, to you know, I couldn't even work out how to reduce the size of my screen, just basics. It is just my abilities from being very assertive and very able to articulate myself. I suddenly almost sort of shrunk as a person. It was quite alarming actually. (Bree)

The suddenness of cognitive changes, taking Bree from being the “go-to” colleague who knew all the work-related policies, to being someone who struggled to remember how to complete basic tasks, was frightening and challenged her sense of self. Being unable to express her ideas and thoughts as clearly as before created a feeling of being less capable (we return to sense of self later). Many women described a feeling of their brains getting “full” more quickly as their processing of information slowed down, which they viewed as another indicator of brain fog.

I don't feel like I can take on as much anymore just because my brain just can't, it just gets too full too quickly and I forget things a lot, so I have to write things down. I'm juggling more anyway so you kind of think, I think the whole thing with work, especially with women, you can blame a lot of things on other things. (Rose)

Looking for explanations was described as a common approach that women take, and in the example above, Rose assumed her cognitive changes were related to the COVID-19 pandemic, particularly the impact of isolation, rather than the menopause. Indeed, many participants had noticed their first menopause symptoms around during COVID, which, upon reflection, they felt were exacerbated by the pandemic: the lifestyle changes imposed by the Government (e.g., lockdown), the impact on work (e.g., fewer staff due to illness), and the overall uncertainty which caused stress.

Difficulties with working and episodic memory not only impacted the women's current working lives, but their career progression, hampering thoughts about future possibilities and jobs they were capable of doing. As Kate described:

It has definitely impacted my work because there have been opportunities to progress and I was too anxious to actually consider doing that. I've never left a job because of how I was feeling, but I have not gone for promotions because I felt I wasn't competent enough to do the job. (Kate)

A perceived decline in competence could lead to increased anxiety among the women, which was further intensified by a vicious cycle of forgetfulness and worry about forgetfulness. This pattern often led them to question their own competence and capabilities, undermining their confidence in their ability to progress their careers.

I'm starting to panic about not remembering and worrying about not remembering, and it seems to dominate a lot of my thoughts and definitely from a career progression point of view you sort of think “I'm at a position where my kids have left home, I've got all this experience and this wealth of knowledge” and people that send me job applications and “oh, you should be getting this”. And I'd say “I can't remember what I had for breakfast, so how the hell can I be applying for this job that's gonna pay me and have this responsibility”… if you feel you can't remember what you did yesterday then you feel like a fraud really and it very much knocks your confidence. (Eve)

Feeling like a fraud, as Eve described, was shared by other participants. It suggests the experience of imposter syndrome; that the women felt they should not be in that professional role, and were not entitled to the responsibility or the wage (more below). Experiencing imposter syndrome in a job they were capable of doing with many years of experience behind them had a substantial impact on the participants' confidence.

### Loss of control and sense of self

The impact of brain fog and memory loss on the women's confidence caused them to feel uncertain about their own behavior. They reported a loss of control, for example as they forgot what they were talking about mid-sentence or the work they had been doing minutes ago, needing to “retrace” their steps. This was very concerning.

I could see that I was not getting as much work done as I should. I'd go off in one direction, I'd forget what I was doing and do something else and then I'd kind of have to retrace my steps. I felt like I was having to do it the next day of work just to make up for the fact that I wasn't really working properly, which I found very frustrating. (Layla)

Having to backtrack and repeat actions previously performed to ensure they had not missed anything, as Layla described, increased the participants' workload. The women could therefore find they were doing more work than before yet feel less capable. The combination of increased workload, cognitive changes, anxiety, and panic attacks had an impact on mental health. Layla went on to say: “*and then suddenly I'd find myself just not recognizing myself”.*

It is telling that many of the women described not recognizing themselves since experiencing these cognitive changes, which indicates the extent of their impact on mental health. Overall, a strong message from the data was that participants felt they were not getting their work done in the same way as before menopause, and this feeling of inefficiency intersected with their sense of identity.

It's like a bereavement. The emotions that you experience, it's a loss, it's because you've lost yourself, basically, and you go through one-thousand-and-one emotions. Yeah, horrendous, horrendous times. (Tess)

The impact on mental health from the suddenness and intensity of these cognitive changes, and the uncertainty about one's own actions, led to doubts in productivity at work, a sense of being out of control, and feeling like they were not themselves. It perhaps comes as no surprise that most participants (*n* = 36) reported feeling overwhelmed at work in the pre-interview questionnaire (data not shown).

### Anxiety, struggle, and adaptation

As indicated above, a sense of inefficiency at work exacerbated the participants' anxiety. Some women experienced anxiety for the first time during the menopause transition, while others with pre-existing anxiety believed that their menopause symptoms had increased it.

I felt like the brain foggy type stuff was starting to make me more anxious. Usually, I'm in a training role and I don't usually get stressed and worried… it's just that bit where your head empties and that's not usually something that happens to me so I was ending up really anxious.. I'm generally quite resilient, I have a lot of things going on just because of my personal life, but yeah, it was sort of getting more difficult. (Phoebe)

Phoebe raised an important point about “things going on” in her personal life and how that could impact her professional experiences. Indeed, a key finding was the overlap between the personal and professional domains of the women's lives, forming a reciprocal relationship in which challenges in one domain influenced experiences in the other. Participants perceived aspects of the work environment to affect the intensity of menopausal symptoms; however, these symptoms did not simply 'switch off' when women moved between work and home. Similarly, sleep disruption associated with menopausal symptoms contributed to fatigue, which participants believed impaired cognitive functioning, including memory and concentration (often described as “brain fog”), with subsequent effects on their work performance.

The reason that I became aware of my chemistry changing was that I was starting to forget significant things at work and my performance was noted to be poorer than it had been, which affected my confidence and brought to my attention that I wasn't sleeping well and that maybe my cognitive abilities were affected sometimes. (Jane)

It was clear that the women could find it a struggle to manage their menopausal symptoms both at home and in their professional lives. A key finding was the process of struggle and adaptation, where participants felt they had to adapt to the “new me” even if it caused them a great deal of frustration.

I was feeling mental confusion, lack of memory, lack of focus, and that's been my major experience all the way through, the hot flushes stopped very quickly with HRT… but since then for 3 1/2 years I've really struggled and had to adapt. I think it's impacted on my job. It's definitely impacted my family and having to get used to myself being unreliable and sort of be frustrated and all these things when I can't do things as efficiently as I normally do. (Layla)

### Masking and workplace support

To avoid being seen as less competent at work, the women would try to hide the impact of their symptoms on their performance. Attempting to “mask” the changes in their abilities in this way could prevent line-managers from seeing any mistakes the women made. This process, alongside concern that they were not “functioning” as they should, was incredibly unsettling for the women.

No one brought it to my attention, so I obviously managed to, you know, fool them that I was still functioning properly, even though I knew I wasn't. (Rachel)

The difficulty in processing information on a regular and long-term basis was frustrating and again presented a challenge to the women's sense of self. Becoming “inefficient” and a fear of being found out connects with the earlier described imposter syndrome.

I felt like within a week or two, my brain was definitely functioning in a way that it was non­functional. Definitely, completely non-functional, to the point of thinking “I'm really not doing my job because I can't think my way through anything”. Usually, my mental processing would be pretty good… it had been going on for months and got so much worse that I was feeling… probably crying every day and just really tearful, just really miserable and most frustrated about not being able to function at work because somebody at some point is going to work out that I'm nonfunctional. (Phoebe)

Masking their reduced performance was a strategy that sometimes worked but sometimes did not. The process of trying to hide their perceived inefficiencies was incredibly stressful. However, it was clear from the findings that certain aspects of the work environment, such as flexible hours, supportive line managers, colleagues who were experiencing similar menopausal symptoms, and the option to work from home, could reduce the impact that menopausal cognitive changes had on women at work. Again, the work environment, when less stressful, could help women deal with their symptoms and take back some control.

I'm very lucky that I've got a female boss, so I can, you know, mention it. I went on a retreat, like a menopause retreat, so it was a day of learning about these things and she gave me the day for free. She said “no that's a wellbeing day, don't take it out of your annual leave because it's what you need and please tell me anything that you've learned”. So that kind of thing is incredibly supportive and being able to talk about it with people at work to say that you can say, “yeah, I'm having a moment, give me a give me a few minutes and I'll be back”. (Rose)

Inflexible and unsupportive work environments were perceived to exacerbate participants' menopausal symptoms. Notably, Isabelle described a lack of support despite her line manager being a woman of a similar age. She attributed this to her manager's apparent lack of understanding, which she believed stemmed from not experiencing menopausal symptoms at all or in the same way. This finding suggests that shared demographic characteristics alone do not necessarily foster empathy or support. Rather, participants perceived that limited awareness and understanding of the diverse experiences of menopause could contribute to unsupportive responses, including referrals to Occupational Health based on concerns about work performance.

The line manager I had at the time, same age as me and from an operational background, when I knew more about it, I did explain it to her. We have kind of menopause awareness sessions and things like that. She didn't know anything about it. She wasn't experiencing any symptoms herself, lucky lady, and I asked for an Occy [occupational] Health referral to see what support is available. She made that referral and then put poor performance as the reason for the referral, and she'd never spoken to me about performance ever… when I had to speak to her about keeping in touch, coming back to work, I remember crying my eyes out on the phone, which I didn't want to do because she didn't understand, she wasn't a sympathetic person, and I remember her trying to be sympathetic, “look, there are things we can do, you could take a demotion”… she might have been saying nice things around it but all I heard was “poor performance” on something that I'd requested when we've never had a performance conversation. (Isabelle)

Sometimes, the conversations with line managers about the difficulties experienced at work were not always a positive experience. For example, it was suggested that Isabelle reduce her working hours or take a demotion. And while her request for a referral to Occupational Health was honored, it seemed incredibly misplaced and distressing to be referred on the basis of “poor performance” when no such discussion about performance had taken place.

Having the opportunity to work flexibly was perceived as part of the solution to help women manage their cognitive challenges, but in practice it was not always effective. Susan, quoted below, worked freelance and thus had flexible working patterns which she could control. However, she still experienced an overwhelming lack of ability to function in all aspects of life, which could result in feelings of hopelessness.

I worked freelance so I can choose to work when I want to. I don't have to phone in sick to anybody, so if I can't go to work I just need to catch up at some other point. But I just couldn't go to work, couldn't drive the car, couldn't do appointments. (Susan)

## Discussion

The findings highlight the pronounced challenges participants went through at work due to menopausal cognitive changes and feeling that their ability to process information had been compromised. Such changes impaired the women's ability to work efficiently, negatively affected their self-confidence, and often changed their professional life-course to avoid career progression opportunities due to fear of being “caught out” with poor performance ratings. Looking at the findings through the lens of Conservation of Resources (10, 11), they indicate menopause as a major life change with demands that can deplete women's cognitive abilities, which in turn negatively impacts their productivity and perceived performance. In many cases, the onset of menopause symptoms was noticed during the COVID-19 pandemic, which arguably compounded the detrimental effects of menopause on mental health, significantly depleting their resources. The lack of such experiences having been considered in existing pandemic preparedness frameworks is of concern.

The sudden onset and heightened severity of cognitive changes during the menopausal transition contributed to uncertainty regarding both current and future occupational productivity. Although participants were aware that memory lapses and brain fog were reported during menopause, the lived experience was more profound than anticipated. These neurocognitive disruptions precipitated feelings of imposter syndrome, illustrating the detrimental psychosocial impact of impaired executive functioning. Feeling out of control, and in a vicious cycle of being forgetful and worrying about being forgetful, was difficult to navigate. The reported loss of confidence in themselves and their ability to perform their roles mirrors that found in other studies (6, 18, 19). This perceived inefficiency could create anxiety for the first time, or exacerbate pre-existing anxiety, highlighting the biopsychosocial nature of the menopause. Such findings illustrate the principle of the resource loss spiral (11), whereby the depleted cognitive resources, such as forgetfulness, can result in further loss of valuable resources, such as mental health. Indeed, data collected from the questionnaire (not presented here) show an increase in mental health issues during the menopause transition for these participants. At the time of the interview, thirty-nine participants reported depression and/or anxiety as menopause symptoms, of which eighteen reported having both depression and anxiety, seventeen reported anxiety alone, and four reported depression alone. Compared with their pre-menopausal mental health histories, where only twelve participants reported depression and/or anxiety, this represents a substantial increase.

A key finding was the profound alteration in identity closely linked to cognitive changes at menopause. Women described a loss of self-confidence due to not being able to perform work tasks at previously sustained levels, despite often possessing years of expertise in their respective fields. This erosion of professional self-concept reflects the complex interplay between neurocognitive decline, psychosocial functioning, and workplace demands, which collectively influence self-perception, perceived competence, and occupational efficacy. Feelings of imposter syndrome, where individuals perceive themselves as inadequate or fraudulent despite evidence of competence, were frequently reported. While global research has documented the negative impact of menopausal symptoms on women's quality of life, there remains a paucity of empirical evidence specifically exploring the intersection between cognitive changes and identity reconstruction within occupational settings. This gap underscores the need for research that examines how cognitive and psychosocial changes during menopause can reshape professional identities and career trajectories (20). Our findings resonate with results from the UK Government's Women's Health Strategy public consultation (14), which highlighted that many women experience a loss of confidence, reduced workplace engagement, and identity disruption linked to unaddressed menopausal symptoms. The consultation revealed that women frequently downplayed or concealed cognitive difficulties to avoid punitive measures such as demotion, thereby compounding psychological distress and perpetuating gendered workplace inequities.

The Conservation of Resources theory posits that individuals confronted with stressors experience a depletion of personal resources such as self-efficacy and social capital (11). This resource loss can trigger a negative spiral unless offset by the acquisition of compensatory resources, such as organisational support and adaptive workplace accommodations. Our findings demonstrate that supportive managerial practices, including empathetic recognition of symptoms and the provision of flexible work arrangements, were instrumental in mitigating the negative cognitive and psychosocial effects of menopause. This is consistent with evidence indicating that supervisory social support substantially improves women's ability to manage menopause challenges at work (6, 20). Conversely, environments characterised by managerial rigidity and lack of awareness exacerbated feelings of inefficiency, leading women to engage in symptom concealment and to sometimes internalise the blame for performance changes. Such organisational cultures were frequently perceived as discriminatory, negatively influencing job satisfaction and long-term career progression. Participants reported a persistent fear of being “found out,” which carried tangible risks such as demotion or enforced reductions in working hours. For women approaching the later stage of their careers, these consequences not only threaten financial security ahead of retirement but also reinforce systemic gender inequalities in the workplace. This has previously been described as a “penalty” women suffer when their ageing bodies “fail to comply” with the norm of an ideal worker (7, 23–25).

Collectively, our findings and corroborating evidence from the UK Government's Women's Health Strategy public consultation, highlight a gap in workplace health strategies. Addressing menopausal cognitive changes requires moving beyond individual-level coping mechanisms to structural, evidence-based interventions that foster identity preservation and create equitable, supportive occupational environments. This shift is key if we are to promote gender equity and mitigate health inequalities professional settings during midlife.

### Health implications

Understanding participants' experiences through the lens of the Conservation of Resources theory highlights that preserving personal resources during the menopause transition is important to supporting women's overall wellbeing (see Figure 2). Cognitive difficulties and unsupportive work environments can create and/or exacerbate mental health symptoms and reduce women's quality of life. From our findings, help for managing menopausal symptoms could include comprehensive cognitive, psychological, and occupational assessments. Integrating cognitive screening and mental health interventions can sustain resource preservation. Comprehensive public health approaches should recognize the diversity of experiences of menopause, and how they can be impacted by pre-existing health conditions such as endometriosis, where pain and fatigue can compromise cognition. They can also help to reframe medical and commercial models which may promote HRT as the panacea for cognitive health: some of our participants who used HRT reported substantial cognitive challenges. The International Menopause Society provides a toolkit for managing cognitive issues in menopause (21).

**Figure 2:**
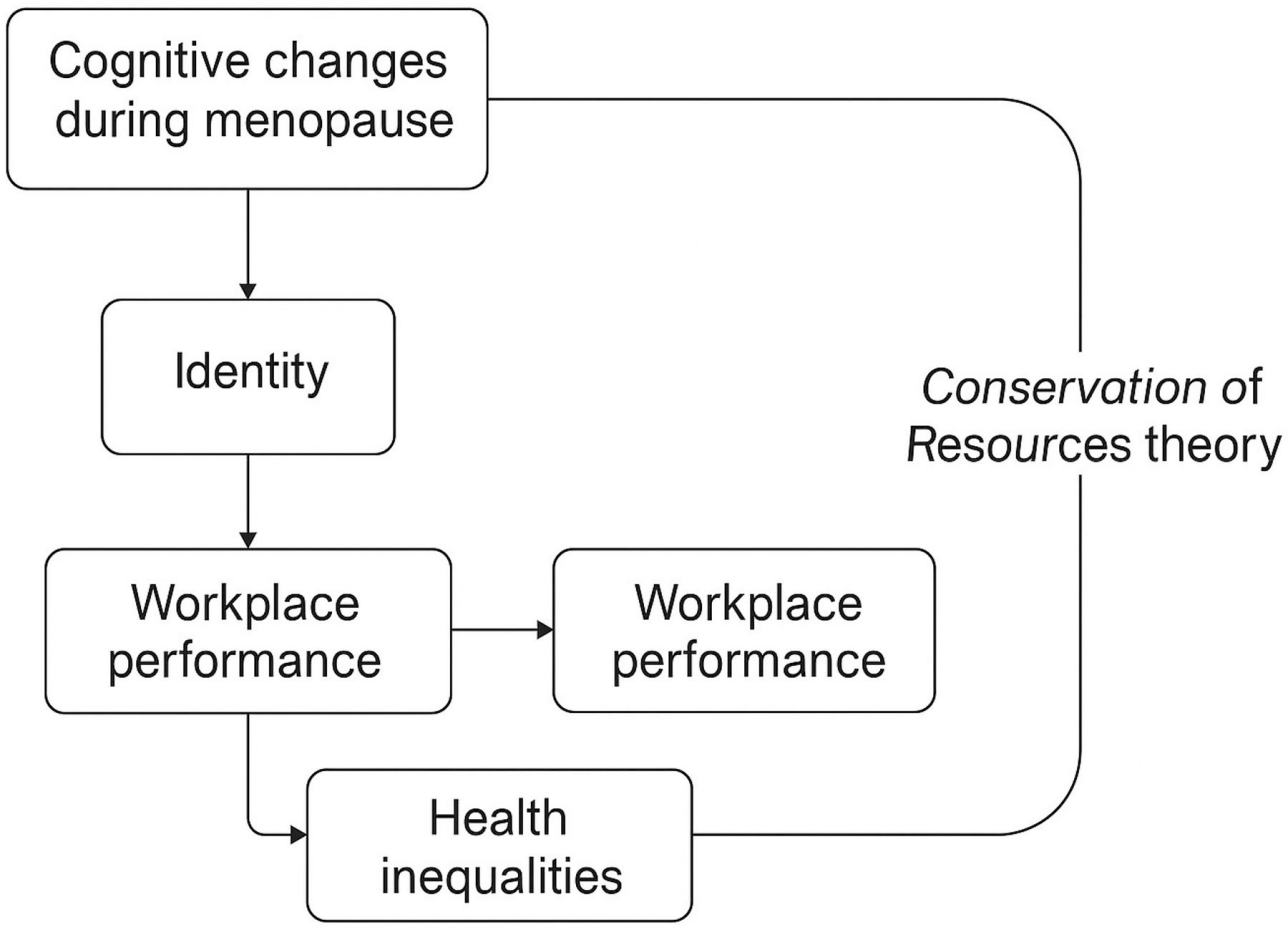
Conceptual diagram illustrating the connection between menopause-related cognitive changes, identity, workplace performance, health inequalities, and the application of conservation of resources.

### Workforce and labour law implications

Menopausal symptoms can impair productivity, self-efficacy, and long-term career trajectories. Organizational culture should evolve to recognize cognitive changes as legitimate occupational health concerns. Indeed, supporting women's wellbeing benefits organizational outcomes by fostering positive work environments and improving productivity. Practical interventions can include autonomy in task management, flexible working patterns, and managerial education. Employers can provide consistent and tangible workplace support by utilizing evidence-based resources like the MARIE toolkit or Menopause Information Pack for Organizations (MIPO).

The UK policy context has continued to develop. The Employment Rights Act 2025 introduced a mechanism through which regulations may require large employers to publish equality action plans addressing the gender pay gap and support for employees experiencing menopause. Employers with at least 250 employees have been able to publish these plans voluntarily since April 2026. Subject to secondary legislation, mandatory publication is intended to commence from spring 2027. Current government guidance identifies actions including workplace adjustments, risk assessment, managerial education and policy review. The findings from our study that inadequate workplace support can exacerbate cognitive and psychological symptoms, leading to reduced job satisfaction and increased risk of demotion or reduced hours, highlights a need for legislative and regulatory reforms that specifically recognize menopause as a workplace health issue. Legal frameworks should mandate employer education, enforce organizational compliance with menopause policies, and incentivize adoption of evidence-based interventions as above. Embedding these changes could mitigate gender inequities and enhance the retention of skilled workers during midlife.

### Strengths and limitations

Conducting a qualitative research study to explore lived experiences has shed light on the ways that cognitive changes at menopause can impact women's professional lives. Our process of data analysis was systematic, adding depth and trustworthiness to the findings. Team-based approaches to qualitative data analysis have been argued to enrich the interpretation of data and conceptual analysis (22). Indeed, our collaborative approach to data analysis was rigorous and revealed rich insights, as four women from different disciplinary backgrounds and age groups interpreted the data. A key strength of our study lies in the broad eligibility criteria, which captured menopausal experiences across cognitive, psychological, and occupational domains like the other qualitative studies of the wider MARIE project. While organisational structures differ, the similarity in participants' experiences suggests that our findings may be transferable to other occupational contexts. We have also captured the perspectives of some who had not sought professional help for their menopause symptoms. In this way, our findings are not biased towards a solely clinical sample and may be applicable to a wider range of women.

The findings are not intended to be statistically generalisable to the UK workforce. The predominantly White, university-educated and professionally employed sample may underrepresent experiences in manual, shift-based, insecure, informal or lower-paid work, where autonomy, flexibility and access to occupational support may differ substantially. Detailed reporting of the sample and study context is provided so that readers can assess the potential transferability of particular findings to comparable settings. As with all qualitative research, there is a potential sample bias as participants self-select, so those who volunteered to take part may in our study may have been particularly motivated to discuss their experiences especially if they were severe or the women felt unheard at work. Indeed, working in the healthcare sector may help people to feel more confident speaking about their health. Unfortunately, the constrained availability of resources restricted our sample size; however, we intend to further diversify our sample by building rapport with a variety of non-governmental organisations and community centres. Menopausal change is not uniform across populations. Thus, conducting further qualitative research inclusive of ethnically diverse and disabled populations is necessary if we are to provide gender-sensitive and shared-care approaches to menopause in the workplace.

## Conclusions

Understanding women's experiences of cognitive changes and mental health in the workplace through the Conservation of Resources theory has shown that menopause is a demand that can substantially deplete women's valuable resources to perform their work duties. Such cognitive changes disrupted their sense of self and perceived ability to work effectively. The intersection of cognitive and psychological symptoms with workplace stressors could exacerbate mental health burdens. Workplace structures often lack appropriate recognition and support for menopausal transitions, which perpetuates gender inequalities. This creates a cycle where women face disproportionate barriers to career advancement, increased risk of economic insecurity, and diminished workplace equity, further entrenching disparities in occupational health and long-term wellbeing. Embedding evidence-based, gender-responsive interventions will protect wellbeing and ensure equitable workforce participation during menopause.

## Data Availability

The raw data supporting the conclusions of this article are not publicly available. Requests for access should be directed to the Principal Investigator of the study Dr. Peter Phiri. Requests will be considered individually, and access may be granted subject to the applicable restrictions and required approvals.
