# Supplementary material for: Understanding the interplay between cognitive changes at menopause, the workplace, and mental health: qualitative findings from the MARIE-UK (WP2a) project
Source: Front Glob Womens Health. 2026 Oct 9;7:1878423. doi: 10.3389/fgwh.2026.1878423 (PMC13693422; doi:10.3389/fgwh.2026.1878423)
Supplement: Supplementary file 1 [file Table1.docx]

**Appendix 1: Consolidated criteria for reporting qualitative studies (COREQ) 32-item checklist**

| **No. Item** | **Guide questions/description** | **Notes** |
| --- | --- | --- |
| **Domain 1: Research team and reﬂexivity** |  |  |
| *Personal Characteristics* |  |  |
| 1. Inter viewer/facilitator | Which author/s conducted the interview or focus group? | GD, JT, SH |
| 2. Credentials | What were the researcher’s credentials? E.g. PhD, MD | All have PhDs |
| 3. Occupation | What was their occupation at the time of the study? | GD: research fellow  JT: professor  SH: professor |
| 4. Gender | Was the researcher male or female? | Female |
| 5. Experience and training | What experience or training did the researcher have? | All have experience of conducting qualitative interviews |
| *Relationship with participants* |  |  |
| 6. Relationship established | Was a relationship established prior to study commencement? | Participants had taken part in the questionnaire part of the study before the interview |
| 7. Participant knowledge of the interviewer | What did the participants know about the researcher? e.g. personal goals, reasons for doing the research | Participants were aware of the aims of the project, the research team explained that the goal was to improve understanding and support for menopause |
| 8. Interviewer characteristics | What characteristics were reported about the interviewer/facilitator? e.g. Bias, assumptions, reasons and interests in the research topic | They were informed that we were experienced researchers with an interest in women’s health |

| **Domain 2: study design** |  |  |
| --- | --- | --- |
| *Theoretical framework* |  |  |
| 9. Methodological orientation and Theory | What methodological orientation was stated to underpin the study? e.g. grounded theory, discourse analysis, ethnography, phenomenology, content analysis | Reflexive thematic analysis,  Conservation of Resources Theory |
| *Participant selection* |  |  |
| 10. Sampling | How were participants selected? e.g. purposive, convenience, consecutive, snowball | Purposive sampling, participants had taken part in the preceding questionnaire past of the study |
| 11. Method of approach | How were participants approached? e.g. face-to-face, telephone, mail, email | An invitation was included at the end of the questionnaire |
| 12. Sample size | How many participants were in the study? | 50 |
| 13. Non-participation | How many people refused to participate or dropped out? Reasons? | Nobody refused to participate or dropped out. 95% of participants signed their consent to be contacted about the qualitative part of the study. The number of those approached for the qualitative part was due to limited resources available |
| *Setting* |  |  |
| 14. Setting of data collection | Where was the data collected? e.g. home, clinic, workplace | Collected online, participants were based either at home or the workplace in private rooms |
| 15. Presence of non-participants | Was anyone else present besides the participants and researchers? | No |
| 16. Description of sample | What are the important characteristics of the sample? e.g. demographic data, date | Age-range 43-67 years (mean 52.3); n=41 were working at the time of interview;  Menopausal status:  menopause *n*=24  post-menopause *n*=14  perimenopause *n*=12  Ethnicity:  white *n*=38)  South Asian *n*=5  white other *n*=4  Caribbean *n*=1  Back American *n*=1  Peruvian *n*=1 |
| *Data collection* |  |  |
| 17. Interview guide | Were questions, prompts, guides provided by the authors? Was it pilot tested? | The interview guide was based on insights gathered from: an evidence synthesis on what is/is not available within the perimenopausal, menopausal and post-menopausal landscape (ie WP1 of the MARIE project); a UK government report published from over 100000 women in the UK that prioritised menopause as a key issue to research; and input from PPI |
| 18. Repeat interviews | Were repeat interviews carried out? If yes, how many? | No |
| 19. Audio/visual recording | Did the research use audio or visual recording to collect the data? | Audio-recorded with participant consent |
| 20. Field notes | Were ﬁeld notes made during and/or after the interview or focus group? | No |
| 21. Duration | What was the duration of the inter views or focus group? | 25-90 minutes |
| 22. Data saturation | Was data saturation discussed? | We believe data saturation was reached, even though our study is not aGrounded Theory study |
| 23. Transcripts returned | Were transcripts returned to participants for comment and/or correction? | No |
| **Domain 3: analysis and ﬁndings** |  |  |
| *Data analysis* |  |  |
| 24. Number of data coders | How many data coders coded the data? | Four |
| 25. Description of the coding tree | Did authors provide a description of the coding tree? | We developed a coding tree and thematic framework, which we discussed regularly to check for shared understanding, and revised as and when required |
| 26. Derivation of themes | Were themes identiﬁed in advance or derived from the data? | Derived from the data |
| 27. Software | What software, if applicable, was used to manage the data? | None |
| 28. Participant checking | Did participants provide feedback on the ﬁndings? | No |
| *Reporting* |  |  |
| 29. Quotations presented | Were participant quotations presented to illustrate the themes/ﬁndings? Was each quotation identiﬁed? e.g. participant number | Yes, quotations are included in the paper, identified by participant pseudonym |
| 30. Data and ﬁndings consistent | Was there consistency between the data presented and the ﬁndings? | All the findings are derived from the dataset |
| 31. Clarity of major themes | Were major themes clearly presented in the ﬁndings? | Major themes derived from the analysis are included in the paper under sub-headings |
| 32. Clarity of minor themes | Is there a description of diverse cases or discussion of minor themes? | Diverse cases were discussed during the analysis process, and included in the paper where relevant |
