# Supplementary material for: Understanding the interplay between cognitive changes at menopause, the workplace, and mental health: qualitative findings from the MARIE-UK (WP2a) project
Source: Front Glob Womens Health. 2026 Oct 9;7:1878423. doi: 10.3389/fgwh.2026.1878423 (PMC13693422; doi:10.3389/fgwh.2026.1878423)
Supplement: Supplementary file 2 [file Table2.docx]

**Appendix 2: Table of themes identified during the data analysis**

| **Main themes** | **Subthemes** |
| --- | --- |
| Menopause symptoms and signs | Mental health  Genito-urinary, sexual wellbeing  Memory, cognition  Tiredness, sleep  Muscular, joints  Vasomotor  Severity  Body, skin  Self-esteem, confidence  Other symptoms  Personality changes |
| Impact of symptoms  7 | Forgetting, fearful, distress  Anxiety  Shame, loss of confidence  Sexual desire loss, GU pain  General: ‘functional wreck’  Always tired  Uncertainty: other health issues |
| Managing the symptoms  10 | Work environment factors  Work environment education / improvement  Appropriate clothing  HRT  Cognitive exercises  Relaxation  Exercise  Diet |
| Reproductive and menstrual history |  |
| HRT use  3 | Using HRT  Not using HRT  Uncertainty about the impact, dealing with side-effects |
| Testosterone use  2 | Tried it  Not tried it |
| Other medications  3 | Anti-depressants  Medicines in general  Alternative remedies |
| Sought help from GP  5 | HRT  Difficult conversations  GP knowledge  Improvements in GP support |
| Help from menopause specialist |  |
| Other sources of help  4 | Talking Therapy, Cognitive Behaviour Therapy  Social circle, friends |
| Social and interpersonal context  6 | Single parent  Covid  Cost of living  Work life balance  Stigma |
| Education needed |  |
| Other health conditions |  |
